# Use of indwelling urinary catheters in nursing home residents: results from a cross-sectional study in 21 German nursing homes

**DOI:** 10.1186/s12894-024-01512-w

**Published:** 2024-06-14

**Authors:** Jonas Czwikla, Guido Schmiemann, Falk Hoffmann

**Affiliations:** 1 Department of Health, Long-term Care and Pensions, SOCIUM Research Center on Inequality and Social Policy, University of Bremen, Mary-Somerville-Straße 5, 28359 Bremen, Germany; 2 High-Profile Area of Health Sciences, University of Bremen, Bibliothekstraße 1, 28359 Bremen, Germany; 3 Department of Health Services Research, School of Medicine and Health Sciences, Carl von Ossietzky Universität Oldenburg, Ammerländer Heerstraße 114-118, 26129 Oldenburg, Germany; 4 Department for Health Services Research, Institute of Public Health and Nursing Research (IPP), University of Bremen, Grazer Straße 4, 28359 Bremen, Germany

**Keywords:** Urinary catheter, Indwelling catheter, Nursing home, Urology, Geriatrics, Health services research

## Abstract

**Background:**

Indwelling urinary catheters often lead to complications such as symptomatic urinary tract infections. In nursing home residents, catheter prevalence is high, but prevalence differences by sociodemographic characteristics, comorbidities, and health services use have rarely been investigated. The purpose of this work was to describe the use of indwelling urinary catheters in nursing home residents and to examine whether catheter use is associated with individual characteristics.

**Methods:**

Cross-sectional data of the “Inappropriate Medication in patients with REnal insufficiency in Nursing homes” (IMREN) study conducted in 21 German nursing homes between October 2014 and April 2015 were analyzed. For all residents of the involved care units, nurses of the participating institutions completed an anonymous questionnaire including the Modified Rankin Scale to assess physical impairments. The proportion of nursing home residents with indwelling urinary catheter was determined. Associations between catheter use and individual characteristics were investigated via cluster-adjusted multivariable logistic regression.

**Results:**

Of 852 residents (76.5% female; mean age 83.5 years), 13.4% had an indwelling urinary catheter. The adjusted odds ratios for catheter use for men vs. women was 2.86 (95% confidence interval 1.82–4.50). For residents with “moderate” disability vs. those with “no to slight” disability it was 3.27 (1.36–7.85), for individuals with “moderately severe” disability vs. the reference group it was 9.03 (3.40–23.97), and for those with “severe” disability vs. the reference group it was 26.73 (8.60–83.14). For residents who had been hospitalized within the last 12 months vs. those without a hospitalization it was 1.97 (1.01–3.87). For age, dementia, overweight/obesity, other indwelling devices, and long-term medications no significant associations were found.

**Conclusions:**

Male nursing home residents, residents with a higher degree of physical impairment, and those who had been hospitalized within the last 12 months were more likely to use an indwelling urinary catheter than their counterparts. Data on circumstances of and indications for catheters, catheter types, and duration of catheterization are needed to evaluate the appropriateness of catheter use in nursing home residents and the need for interventions.

## Background

The use of indwelling urinary catheters can lead to symptomatic urinary tract infections and other complications, such as catheter blockage, urine leakage, and urethral trauma [1–3]. Furthermore, indwelling urinary catheters are often used without clear indications and inappropriately, which is especially common in older adults [4–6]. Guidelines from the United States of America [7–9], United Kingdom [10], and China [11], among others, recommend to insert indwelling urinary catheters only for appropriate indications, including acute urinary retention, urinary obstruction, urine output monitoring in critically ill patients, selected surgical procedures, assistance in wound healing for incontinent individuals, and comfort care in terminally ill persons [7, 8, 11]. They also recommend to remove placed catheters as soon as possible [7–11], to consider alternatives for indwelling catheterization such as external and intermittent catheters where possible [7–11], and to not use indwelling urinary catheters for the management of incontinence [7, 9, 11].

In nursing homes, many residents have indications for indwelling urinary catheters and the catheter prevalence is higher than in the general population [12] or among home care recipients [13]. In a recent systematic review including 67 studies, the catheter prevalence in nursing home residents ranged from 2.2 to 36.4%, with a median of 7.3% [14]. Furthermore, the prevalence varied between countries and was comparatively high in Germany, ranging from 7.3 to 28.0%, with a median of 10.2% [14]. However, because the majority of existing studies did not focus exclusively on catheters, they did not report on prevalence differences by individual characteristics of residents. Even for differences by sex, only nine studies [15–23] could be included in the review and for differences by age, only one study [22] was included [14]. Whether certain groups of residents are more likely to be catheterized than others is therefore still unclear.

The purpose of this work was (i) to describe the use of indwelling urinary catheters in nursing home residents, and (ii) to investigate whether indwelling urinary catheter use is associated with sex, age, physical impairments, dementia, overweight/obesity, hospitalizations, the use of other indwelling devices, and long-term medications.

## Methods

### Study design and data source

This work was conducted using data from the “Inappropriate Medication in patients with REnal insufficiency in Nursing homes” (IMREN) study, which is described in detail elsewhere [24–27]. In brief, the multicenter cross-sectional IMREN study was carried out in Bremen (urban region) and the surrounding area of Lower Saxony (predominantly rural region) between October 2014 and April 2015. It included a convenience sample of 21 nursing homes, which agreed to participate and were heterogenous in terms of sponsorship, location, and size. Twenty-one nursing homes were included because according to a sample size calculation, 856 residents from 19 nursing homes (45 per facility) were needed to precisely estimate the prevalence of renal failure in nursing home residents, which was a primary aim of the original IMREN study [24]. An anonymized data collection which included all residents of the involved care units (i.e., no exclusion criteria were applied) was conducted by the nurses of the participating nursing homes. The nurses completed a piloted two-page questionnaire based on a review of existing nursing records, and provided a copy of the current medication schedule for each resident (i.e., residents themselves were not involved). Besides information on medication and renal insufficiency, the collected data comprised information on sociodemographic characteristics, comorbidities and health services use. To ensure anonymity of the collected data, the questionnaire included no questions on person identifiers, and the nurses anonymized the provided copies of medication schedules according to specific instructions. All nurses were trained in how to collect the data and a monetary incentive was provided for each completed questionnaire.

The study was approved by the University of Bremen Ethics Committee (reference number “IMREN”). To guide reporting of this work, we followed the STROBE statement [28].

### Outcome and covariates

Data on the use of indwelling urinary catheters (outcome) were used to differentiate between residents with / without an indwelling urinary catheter at the time of data collection. The information was assessed using the question “Does the resident currently have an indwelling urinary catheter?” (response options “No” and “Yes”; i.e., no differentiation regarding the type of catheter was made).

For covariates, besides data on sex and age, information on physical impairments at the time of data collection were assessed using the Modified Rankin Scale including six grades [29]. These are: “0 no symptoms at all”, “1 no significant disability despite symptoms: able to carry out all usual duties and activities”, “2 slight disability: unable to carry out all previous activities but able to look after own affairs without assistance”, “3 moderate disability: requiring some help, but able to walk without assistance”, “4 moderately severe disability: unable to walk without assistance, and unable to attend to own bodily needs without assistance”, and “5 severe disability: bedridden, incontinent, and requiring constant nursing care and attention”. It was applied using the instruction “Please indicate which of the descriptions reflects the current grade of physical impairments of the resident best (only one answer please).”. For this work, grades 0–2 were collapsed because only few nursing home residents had “no to slight” disability. Furthermore, information on dementia were assessed by asking the nurses whether the resident had ever been diagnosed with dementia (response options “No” and “Yes”). Data on weight in kg and height in m of the resident were also provided by the nurses to calculate the body mass index (BMI) (weight divided by the square of the height). Data on hospitalizations were measured using the question “Has the resident been in a hospital at least once in the past 12 months?” (response options “No” and “Yes”). Information on feeding tubes and bowel stomas were gathered using the questions “Does the resident currently have a feeding tube?” and “Does the resident currently have a bowel stoma?” (response options “No” and “Yes”). Data on long-term medications (i.e., the number of regularly prescribed drugs, excluding pro re nata and short-term medication) were extracted from medication schedules and served as a proxy for comorbidities [30].

### Statistical analysis

In the first step, the characteristics of nursing home residents were analyzed in total and separately for both sexes. The distributions of age groups (< 65, 65–74, 75–84, 85 + years) and physical impairments (“no to slight”, “moderate”, “moderately severe”, “severe” disability), the prevalence of dementia, the distribution of the BMI (< 25 [no overweight/obesity], 25–29 [overweight], 30 + [obesity]), the prevalence of hospitalizations within the last 12 months and feeding tubes/bowel stomas (feeding tube and/or bowel stoma, feeding tube, bowel stoma), and the distribution of long-term medications (0–4, 5–9, 10 + drugs) were determined. Furthermore, the mean and median age, BMI, and number of long-term medications were calculated.

Second, the proportion of nursing home residents with indwelling urinary catheter was determined for all residents and separately for women and men. These calculations were also conducted by age group, physical impairment, dementia, BMI category, hospitalizations within the last 12 months, feeding tube and/or bowel stoma, and the categorized number of long-term medications, whereby the same categorizations were applied as in step one of the analysis. Statistically significant differences were identified based on 95% confidence intervals (CIs), which were adjusted for cluster sampling because hierarchical data from residents clustered in nursing homes were analyzed.

In the third step, a cluster-adjusted multivariable logistic regression was applied to investigate which individual characteristics examined in step two of the analysis are associated with the use of indwelling urinary catheters. In this regression, indwelling urinary catheter use served as the dependent variable. The independent variables were sex, age group, physical impairment, dementia, BMI category, hospitalization, feeding tube and/or bowel stoma, and the categorized number of long-term medications. Statistically significant associations were identified based on odds ratios, 95% CIs, and p-values < 0.05. Goodness of fit of the model was assessed using the c-statistic.

All analyses were performed using SAS 9.4 (SAS Institute Inc., Cary, NC, USA), taking into account procedures for complex survey designs.

## Results

The study included 852 residents (mean time of residence 3.2 years [median 2.1 years]) from 21 nursing homes (57.1% independent nonprofit nursing homes and 42.9% privately sponsored nursing homes; 52.4% nursing homes from Bremen and 47.6% nursing homes from the surrounding area of Lower Saxony). More than every third resident (76.5%) was female, and the mean age was 83.5 years (Table 1). Of all residents, 82.6% had “moderate”, “moderately severe”, or “severe” disability, and 57.7% suffered from dementia. The prevalence of overweight and obesity was 31.0% and 16.1%, respectively. Within the last 12 months, 43.1% of residents were hospitalized, and at the time of data collection, 5.4% had a feeding tube and/or bowel stoma. The mean number of long-term medications was 6.3.

Stratified by sex, the mean age and the prevalence of dementia were higher in women than in men (85.0 vs. 78.3 years and 59.5% vs. 51.0%), whereas the prevalence of overweight and hospitalizations were lower in women than in men (27.7% vs. 42.0% years and 40.3% vs. 52.4%). The distribution of physical impairments as well as the prevalence of obesity and feeding tubes and/or bowel stomas differed only slightly by sex. This was also true for the mean number of long-term medications.

**Table 1:** Characteristics of nursing home residents in total and by sex

Category	Women (*n* = 647; 76.5%) %	Men (*n* = 199; 23.5%) %	Total (*n* = 852; 100%) %
**Age group**			
**(*n*** = **647 women, 199 men, 852 total)**			
< 65 years	4.2	12.1	6.0
65–74 years	5.3	17.6	8.1
75–84 years	26.9	36.2	29.1
85 + years	63.7	34.2	56.8
Mean (SD); Median (IQR)	85.0 (9.7); 87.0 (81.0–91.0)	78.3 (11.4); 80.0 (72.0–87.0)	83.5 (10.5); 86.0 (79.0–91.0)
**Physical impairment**			
**(*n*** = **641 women, 194 men, 841 total)**			
no to slight disability	16.4	20.1	17.4
moderate disability	33.4	27.3	31.9
moderately severe disability	30.7	29.4	30.4
severe disability	19.5	23.2	20.3
**Dementia**			
**(** ***n*** ** = 635 women, 196 men, 837 total)**			
Yes	59.5	51.0	57.7
**Body mass index**			
**(** ***n*** ** = 629 women, 193 men, 828 total)**			
< 25 (no overweight/obesity)	55.2	45.1	52.9
25–29 (overweight)	27.7	42.0	31.0
30 + (obesity)	17.2	13.0	16.1
Mean (SD); Median (IQR)	24.9 (5.5); 24.2 (21.1–27.7)	25.7 (5.5); 25.3 (22.1–28.1)	25.1 (5.5); 24.5 (21.3–27.9)
**Hospitalization within the last 12 months**			
**(** ***n*** ** = 613 women, 191 men, 810 total)**			
Yes	40.3	52.4	43.1
**Feeding tube/bowel stoma**			
**(*n*** = **641 women, 198 men, 845 total)**			
Feeding tube and/or bowel stoma	4.8	7.6	5.4
feeding tube	3.4	6.6	4.1
bowel stoma	1.6	1.0	1.4
**Number of long-term medications**			
**(** ***n*** ** = 647 women, 199 men, 852 total)**			
0–4 drugs	30.6	30.2	30.3
5–9 drugs	51.8	55.8	52.7
10 + drugs	17.6	14.1	17.0
Mean (SD); Median (IQR)	6.3 (3.4); 6.0 (4.0–9.0)	6.2 (3.1); 6.0 (4.0–8.0)	6.3 (3.4); 6.0 (4.0–9.0)

Abbreviations: SD, standard deviation; IQR, interquartile range

### Proportions with indwelling urinary catheter

Of all residents, 13.4% (9.7% of women and 25.3% of men) had an indwelling urinary catheter (Table 2). Although statistically not significant, the catheter prevalence tended to decrease with increasing age from 33.3% in residents aged < 65 years to 8.6% in 85 +-year-old residents. With respect to physical impairments, catheter prevalence increased from 2.1% in residents with “no to slight” disability to 32.4% in severely disabled residents. The prevalence tended to be lower in residents suffering from dementia compared to those without dementia but the difference of 10.7% vs. 16.7% was not significant. A significantly higher prevalence was observed in residents who had been hospitalized within the last 12 months compared to those without a hospitalization (19.0% vs. 9.0%), and in residents who had a feeding tube and/or bowel stoma at the time of data collection compared to those without such an indwelling device (43.5% vs. 11.6%). Regarding the BMI categories and categorized number of long-term medications, almost no prevalence differences were observed.

Stratified by sex, the catheter prevalence decreased with increasing age in women (from 29.6% [< 65 years] to 6.9% [85 + years]) and men (from 37.5% [< 65 years] to 17.6% [85 + years]), but only in women was the decrease statistically significant. With regard to physical impairments, the prevalence increased from 0.0% in women and 7.7% in men with “no to slight” disability to 28.2% and 44.4% in severely disabled women and men. In particular in men, the catheter prevalence tended to be lower in individuals suffering from dementia compared to those without dementia (17.2% vs. 32.3%). Regarding BMI, no significant differences were observed in both sexes. Women who had been hospitalized within the last 12 months had a significantly higher prevalence than those without a hospitalization (14.6% vs. 6.1%), whereas in men the difference was not significant. Women and men who had a feeding tube and/or bowel stoma at the time of data collection had a higher prevalence than those without such a device (38.7% vs. 8.2% [women] and 53.3% vs. 23.0% [men], respectively) but only in women was the difference significant. Regarding long-term medications, the catheter prevalence tended to increase from 15.0% in men treated with 0–4 drugs to 32.1% in men treated with 10 + drugs, but the increase was not significant.

**Table 2:** Proportion of nursing home residents with indwelling urinary catheter in total and by sex

Category	Women (*n* = 641)		Men (*n* = 198)		Total (*n* = 845)	
	%	95% CI	%	95% CI	%	95% CI
**Total**						
**(** ***n*** ** = 641 women, 198 men, 845 total)**	9.7	6.9–12.4	25.3	17.8–32.7	13.4	9.9–16.9
**Age group**						
**(** ***n*** ** = 641 women, 198 men, 845 total)**						
< 65 years	29.6	14.6–44.7	37.5	0.0-77.1	33.3	9.1–57.5
65–74 years	11.8	0.0–26.0	38.2	26.8–49.7	25.0	15.2–34.8
75–84 years	12.7	6.7–18.7	22.2	11.5–32.9	15.4	8.9–21.8
85 + years	6.9	4.4–9.4	17.6	7.3–28.0	8.6	5.9–11.3
**Physical impairment**						
**(** ***n*** ** = 635 women, 193 men, 834 total)**						
no to slight disability	0.0	NA	7.7	0.1–15.3	2.1	0.0-4.3
moderate disability	2.8	0.9–4.8	15.4	6.5–24.3	5.7	2.7–8.6
moderately severe disability	10.8	5.3–16.3	31.6	19.4–43.8	15.4	9.8–21.0
severe disability	28.2	21.0-35.4	44.4	23.4–65.5	32.4	24.0-40.7
**Dementia**						
**(** ***n*** ** = 630 women, 195 men, 831 total)**						
No	10.9	7.3–14.5	32.3	21.1–43.4	16.7	11.6–21.7
Yes	8.8	5.8–11.9	17.2	11.9–22.4	10.7	7.7–13.7
**Body mass index**						
**(** ***n*** ** = 624 women, 192 men, 822 total)**						
< 25 (no overweight/obesity)	9.9	6.5–13.4	30.2	19.7–40.7	14.1	9.3–18.9
25–29 (overweight)	8.1	1.3–14.9	18.5	8.1–28.9	11.3	4.1–18.5
30 + (obesity)	13.0	5.4–20.5	32.0	10.6–53.4	16.5	8.9–24.2
**Hospitalization within the last 12 months**						
**(** ***n*** ** = 607 women, 190 men, 803 total)**						
No	6.1	3.0-9.2	20.9	10.9–30.9	9.0	4.9–13.1
Yes	14.6	10.1–19.1	29.3	19.8–38.7	19.0	14.0-24.1
**Feeding tube and/or bowel stoma**						
**(** ***n*** ** = 641 women, 198 men, 845 total)**						
No	8.2	5.3–11.1	23.0	15.8–30.1	11.6	8.1–15.2
Yes	38.7	17.8–59.6	53.3	23.0-83.7	43.5	24.9–62.0
**Number of long-term medications**						
**(** ***n*** ** = 641 women, 198 men, 845 total)**						
0–4 drugs	9.1	4.4–13.8	15.0	4.5–25.5	10.5	6.0-14.9
5–9 drugs	10.0	5.9–14.1	29.1	18.9–39.3	14.6	10.0-19.2
10 + drugs	9.8	2.6–17.1	32.1	8.5–55.8	14.7	5.6–23.8

Abbreviations: CI, confidence interval; NA, not applicable

### Multivariable analysis

The multivariable logistic regression confirmed that male sex, a higher degree of physical impairment, and being hospitalized within the last 12 months was positively associated with indwelling urinary catheter use (Table 3). The adjusted odds ratio for catheter use for men vs. women was 2.86 (95% CI 1.82–4.50). For residents with “moderate” disability vs. those with “no to slight” disability it was 3.27 (1.36–7.85), for individuals with “moderately severe” disability vs. the reference group it was 9.03 (3.40-23.97), and for those with “severe” disability vs. the reference group it was 26.73 (8.60-83.14). For residents who had been hospitalized within the last 12 months vs. those without a hospitalization it was 1.97 (1.01–3.87). Regarding dementia, a tendency for a negative association with catheter use was observed. The adjusted odds ratio for catheter use for individuals suffering from dementia vs. those without dementia was 0.62 (0.37–1.04). With respect to age, BMI, feeding tubes and/or bowel stomas, and long-term medications no associations were observed. The c-statistic was 0.825 indicating a strong predictive power of the model.

**Table 3:** Multivariable logistic regression for the probability of having an indwelling urinary catheter

Variable	Total		
	(*n* = 758)		
	OR	95% CI	*p*-value
**Sex**			
Male (ref. female)	**2.86**	**1.82–4.50**	**0.0001**
**Age group (ref. <65 years)**			
65–74 years	0.88	0.26–3.02	0.8263
75–84 years	0.91	0.31–2.67	0.8636
85 + years	0.60	0.19–1.87	0.3588
**Physical impairment (ref. no to slight disability)**			
moderate disability	**3.27**	**1.36–7.85**	**0.0106**
moderately severe disability	**9.03**	**3.40-23.97**	**0.0001**
severe disability	**26.73**	**8.60-83.14**	**< 0.0001**
**Dementia**			
Yes (ref. no)	0.62	0.37–1.04	0.0668
**Body mass index (ref. <25 [no overweight/obesity])**			
25–29 (overweight)	0.89	0.32–2.44	0.8057
30 + (obesity)	1.58	0.77–3.25	0.2030
**Hospitalization within the last 12 months**			
Yes (ref. no)	**1.97**	**1.01–3.87**	**0.0477**
**Feeding tube and/or bowel stoma**			
Yes (ref. no)	1.73	0.53–5.63	0.3409
**Number of long-term medications (ref. 0–4 drugs)**			
5–9 drugs	1.56	0.97–2.53	0.0663
10 + drugs	1.36	0.49–3.78	0.5326

Abbreviations: OR, odds ratio; CI, confidence interval; ref., reference

*Note* Boldface indicates statistical significance

## Discussion

This work examined the use of indwelling urinary catheters in 21 German nursing homes and found a catheter point prevalence of 13.4%. Catheter use was associated with male sex, a higher degree of physical impairment, and being hospitalized within the last 12 months. Furthermore, residents suffering from dementia tended to use catheters less frequently than those without dementia. Catheter use was not associated with age, overweight/obesity, other indwelling devices, and long-term medications.

### Catheter use by sex and age

The overall catheter prevalence of 13.4% is higher than the median prevalence of 7.3% reported in a recent systematic review including 67 studies from 21 countries [14]. It is also higher than the median prevalence of available studies from Germany (10.2%; *N* = 15), the US (9.3%; *N* = 9), UK (6.9%; *N* = 7), and Sweden (7.3%, *N* = 6), but in the range of 7.3–28.0% expectable based on the existing German studies [14]. Regarding differences in the urinary catheter prevalence by sex, we observed a higher catheter prevalence in male compared to female nursing home residents, which is in line with other German studies [17, 20, 21] and was also the case in residents from Italy [15] and Sweden [18, 19]. It is also in line with a more than 30-year old study from the US [23], but another American study conducted in the 1980s reported the opposite [22]. In our study the absolute prevalence difference between men and women was 15.6% points, and in the existing studies with a higher prevalence in men it ranged from 5.4 [15] to 27.6 [20] percentage points [14]. From a clinical practice perspective, the prevalence difference might be explained by a higher need for catheters in men resulting from urinary retention, which predominantly affects men [31]. However, it remains unclear whether the total difference in prevalence reflects different needs due to underlying diseases or if male residents are more likely to have inappropriate catheters. Furthermore, it needs to be considered that the catheter prevalence in nursing home residents is higher for transurethral catheters than for suprapubic catheters [14] and that prevalence differences between women and men might vary by catheter type [20, 22].

With respect to prevalence differences by age, we found no significant association with catheter use, probably because our study population was too small. In our descriptive analysis, however, the catheter prevalence in women decreased by age and in men tended to decrease with increasing age. This would be comparable with a more than 30-year old American study [22], which was the only study of 67 studies included in a recent systematic review reporting catheter prevalence in nursing home residents by age [14]. Overall, the evidence regarding prevalence differences by age remains inconclusive.

Given our results on catheter use by sex and age, future studies should include a larger number of nursing home residents, stratify their analyses by sex, and examine whether the catheter prevalence decreases with increasing age. Furthermore, they should collect data on indications for catheters and catheter types to determine proportions of catheterized residents with appropriate indications for transurethral/suprapubic catheters in both sexes and evaluate the need for sex-specific interventions. In countries like Germany, this would also help to identify reasons for the comparatively high catheter prevalence in nursing homes, which is necessary given the limited number of appropriate indications for indwelling urinary catheters mentioned in international guidelines [7, 8, 11] and comparable national recommendations [32], and the high risk for catheter-related complications [1–3].

### Catheter use by physical impairments and dementia

With regard to physical impairments, we found that the catheter prevalence increased with increasing disability to a maximum of 28.2% and 44.4% in severely disabled female and male residents, respectively. This has not been reported before and could be explained from a practical point of view by a large proportion of severely disabled residents who were critically or terminally ill and needed a catheter for urine output monitoring or comfort care [7, 8, 11]. Furthermore, it should be considered that the definition of *severe disability* used in the Modified Rankin Scale includes *incontinence* [29]. It can therefore be assumed that nearly all incontinent residents who had a catheter for the assistance in wound healing [7, 8, 11] were classified as severely disabled. However, it remains unclear whether the total prevalence differences by physical impairments reflect different needs or whether the risk for inappropriate catheters increases with increasing disability.

Regarding dementia, we observed no significant association with catheter use, which is in line with an Italian study [15]. However, in our descriptive analysis the prevalence in individuals suffering from dementia was 6.0% points lower compared to those without dementia, whereby the prevalence difference in women and men was 2.1 and 15.1% points, respectively. A reason for the prevalence difference might be that indwelling urinary catheters require daily care [7, 8, 10, 11], which is more difficult to provide in residents with dementia compared to those without dementia.

Overall, further studies are needed to investigate the appropriateness of urinary catheters in female and male residents with different grades of physical impairments. This is particularly true given that according to international guidelines and national recommendations, physical impairments in nursing home residents usually are not an appropriate indication for indwelling urinary catheters [7, 8, 11, 32]. Furthermore, urinary catheters should not be used as a substitute for nursing care [7, 9, 11, 32]. Studies that explore potential differences in the catheter prevalence by dementia would also be useful to improve our understanding of their use in this growing population.

### Catheter use by hospitalizations

With respect to hospitalizations, we showed that residents who had been hospitalized within the last 12 months were more likely to be catheterized than those without a hospitalization. However, when interpreting this result, it needs to be considered that we were unable to examine whether residents with a catheter were catheterized before, during, or after hospitalization. Existing studies showed that, on the one hand, catheter-related complications in Chinese nursing home residents often lead to hospitalizations [33] and that, on the other hand, many catheterized residents in Germany, the UK, and Sweden received their catheters in a hospital [18, 34–36]. The international literature also shows that male nursing home residents are more likely to be hospitalized than female residents [37], that male (vs. female) residents in Sweden are more likely to be catheterized long term [18, 19], and that in the general population of hospitalized patients in the US, Italy, and Korea, the risk for inappropriate catheters increases with the duration of catheterization [38–40].

Overall, it might be possible that some catheters in nursing home residents who were catheterized in a hospital and/or are catheterized long term are no longer needed. This is especially true since there is evidence that interventions like stop orders, prompting physicians or nurses to remove catheters by default under certain conditions, can reduce the prevalence of inappropriate catheters in hospitalized patients [41]. Studies should examine whether the proportion of catheterized female/male residents with appropriate catheter indications depends on the place where the residents received their catheters and the duration of catheterization. Furthermore, they should investigate whether communication between hospitals and nursing homes during care transitions can be improved (e.g., regarding catheter indications) and whether the catheter need in catheterized residents is continuously evaluated.

### Strengths and limitations

An important strength of this work is that the prevalence of indwelling urinary catheters in nursing home residents was investigated by many individual characteristics which have rarely been examined before. A second strength is that the data on catheterization and covariates are likely to be valid because they were obtained from the nurses in the participating nursing homes who know the residents and reviewed the nursing records to answer the questionnaire. Further strengths are the heterogenous sample of nursing homes; there were no selection criteria for nursing homes in terms of specific diseases (like facilities specialized in neurological diseases) and all residents of the participating care units were included.

At the same time, there are some important limitations to consider. First, we analyzed cross-sectional data including no information on indications for catheterization, catheter types, the place where residents received their catheters, and duration of catheterization. Therefore, we were unable to establish causality, evaluate whether the catheter use was appropriate, and investigate the association between catheter use and hospitalizations in more detail. However, most of the 67 studies included in a recent systematic review did not have the mentioned information either [14]. Second, the questionnaires were answered by nurses in the participating nursing homes and it cannot be ruled out that some questions were answered based on memory rather than scrutiny of records. However, there is no evidence that our results are limited by differential misclassification. Third, we analyzed data assessed almost a decade ago. However, most existing studies in the nursing home setting have not reported differences in catheter prevalence by individual characteristics of residents [14], which is one reason why we reanalyzed our data. Furthermore, there are no indications that catheter use in German nursing homes has changed in the meantime. Fourth, because this study used a convenience sample of nursing homes located in northwest Germany and willing to participate, the generalizability of our results might be limited. For example, catheter use might differ between the West of Germany and the East (i.e., the former German Democratic Republic) due to historical reasons. However, we expect no selection bias at the resident level because all residents of the participating care units were included and our proportions of female and 85 +-year-old residents are comparable with nationwide data (77% vs. 72% female residents and 57% vs. 51% 85 +-year-old residents) [42]. We also expect no selection bias at the nursing home level because a heterogenous sample of nursing homes was included and the sponsorship distribution was also comparable with nationwide data (57% vs. 53% independent nonprofit nursing homes and 43% vs. 42% privately sponsored nursing homes) [42]. Furthermore, both urban and rural nursing homes of various sizes were included. Nevertheless, the limited geographic variability of our study remains an important limitation, and our findings may not be generalizable to countries with a considerably higher/lower catheter prevalence and/or other health and long-term care systems. For instance, the catheter prevalence in nursing home residents varies in Europe from 0.8% in Lithuania to 19.4% in Poland [43], whereby the proportion of nursing home residents among all older people is 10.3% and 0.8% in these two countries [44]. Finally, our findings may not be generalizable to the general population of community dwelling individuals or home care recipients because in these population groups catheter prevalence is lower than in the nursing home setting [12, 13].

## Conclusions and implications

This work showed that in German nursing homes, male residents, residents with a higher degree of physical impairment, and residents who had been hospitalized within the last 12 months were more likely to use an indwelling urinary catheter than their counterparts. Furthermore, catheter use tended to be negatively associated with dementia and might be negatively associated with age. Taking these findings and the results of a recent systematic review [14] into account, future studies on catheters in nursing home residents are warranted. They should stratify their analysis by sex, and investigate potential prevalence differences by age and dementia. Furthermore, studies are needed that collect data on circumstances of and indications for catheters, catheter types, and duration of catheterization to evaluate the appropriateness of catheter use in nursing home residents with a special focus on men, severely disabled residents, and those who have been hospitalized. If these studies find that inappropriate catheter use in nursing home residents is common, interventions to reduce unnecessary catheters are needed.

## Data Availability

The data that support the findings of this study are available from the KfH-Stiftung Präventivmedizin but restrictions apply to the availability of these data, which were used under license for the current study, and so are not publicly available. Data are however available from the authors upon reasonable request and with permission of the KfH-Stiftung Präventivmedizin.

## Abbreviations

BMI: Body Mass Index
CI: Confidence Interval
IMREN: Inappropriate Medication in patients with REnal insufficiency in Nursing homes
